# Invasive papillary carcinoma treated with neoadjuvant endocrine therapy in which pathological complete response was achieved

**DOI:** 10.1186/s13104-016-1854-4

**Published:** 2016-01-26

**Authors:** Chiaki Saita, Risa Goto, Tomoyuki Aruga, Nami Idera, Yayoi Honda, Kazumi Horiguchi, Hiromi Miyamoto, Shinichiro Horiguchi, Toshinari Yamashita, Katsumasa Kuroi

**Affiliations:** Department of Surgery, Tokyo Metropolitan Cancer and Infectious Diseases Center Komagome Hospital, 3-18-22 Honkomagome, Bunkyo-ku, Tokyo, 113-8677, Japan; Department of Pathology, Tokyo Metropolitan Cancer and Infectious Diseases Center Komagome Hospital, 3-18-22 Honkomagome, Bunkyo-ku, Tokyo, 113-8677, Japan

**Keywords:** Breast cancer, Neoadjuvant endcrine therapy

## Abstract

**Background:**

Invasive papillary carcinoma is a rare type of invasive ductal carcinoma. Neoadjuvant endocrine therapy is now considered as an optional therapy for postmenopausal women with hormone receptor-positive breast cancers, including invasive papillary carcinoma.

**Case presentation:**

We discuss the case of an 83-year-old postmenopausal Japanese female with hormone receptor-positive invasive papillary carcinoma who started treatment with an aromatase inhibitor and achieved pathological complete response after 12 months of endocrine treatment.

**Conclusion:**

Appropriate drugs and durations of neoadjuvant endocrine treatment have yet to be established. Continuing therapy with an aromatase inhibitor until the best clinical response is achieved may represent one of the best strategies in neoadjuvant endocrine therapy.

## Background

Invasive papillary carcinoma is a rare type of invasive ductal carcinoma, defined as having a predominantly papillary morphology (>90 %) in the invasive component [1]. Neoadjuvant endocrine therapy is now considered an optional therapy for postmenopausal woman with hormone receptor-positive breast cancer, but achievement of pathological complete response (pCR) is relatively rare.

## Case presentation

**Fig. 1:**
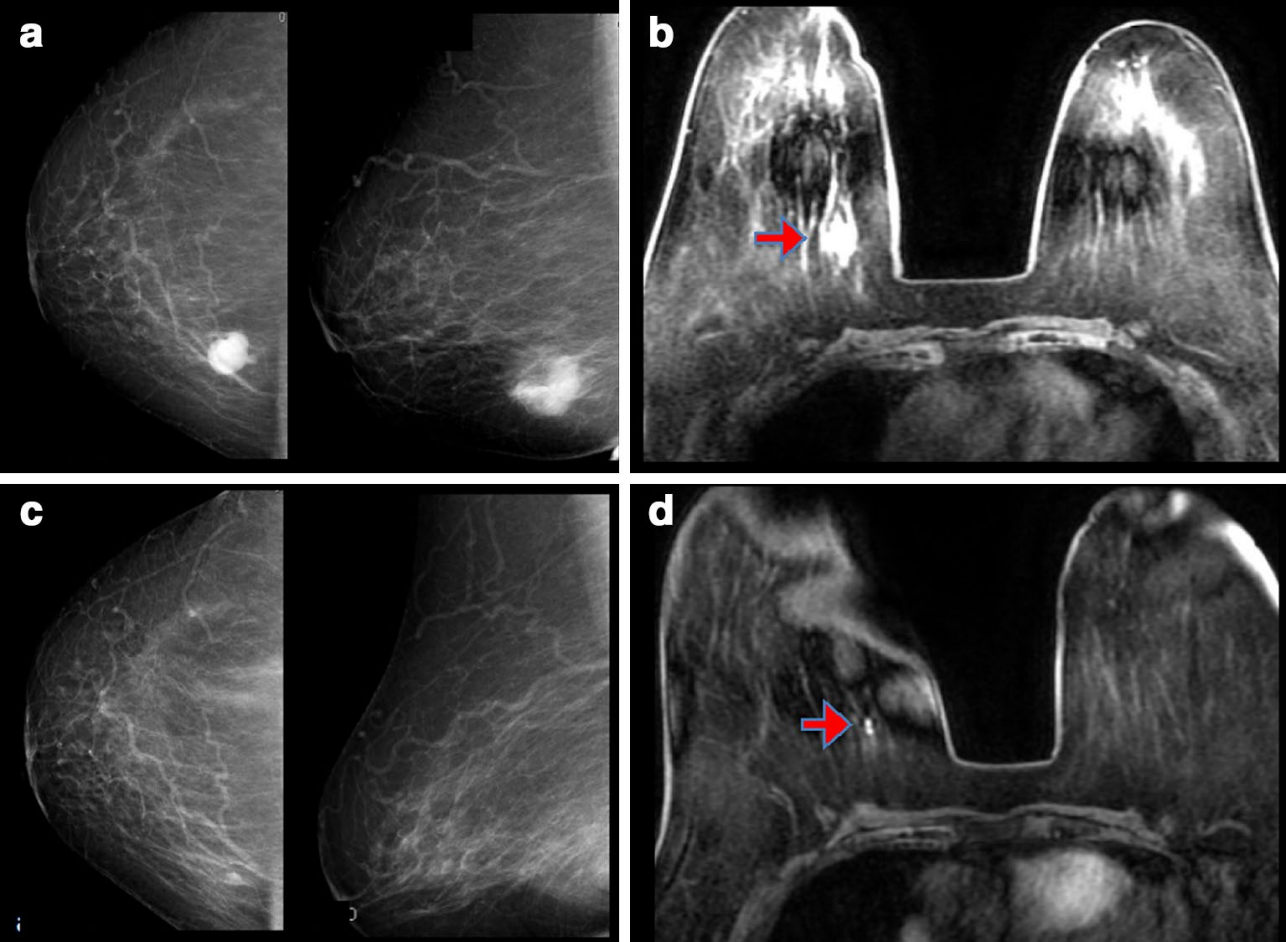
Radiologic appearance of the tumor. **a** Mammography shows a dense mass with partially indistinct margin in the right breast. **b** Contrast-enhanced MRI shows a high-intensity lesion on T2- and diffusion-weighted imaging. **c**, **d** After 12 months of letrozole treatment, mammography reveals disappearance of the tumor **(c)** and MRI shows no evidence of tumor (**d)**

**Fig. 2:**
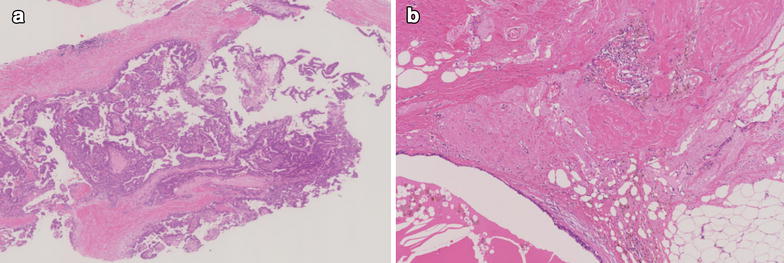
Histological findings of the biopsy and breast conserving surgery. **a** Histopathological examination reveals proliferation of cancer cells with papillary structures, accounting for over 90 % of the whole lesion, supported by arborizing fibrovascular stroma (hematoxylin-eosin). **b** Pathological examination shows fibrosis and elastosis, which was thought to represent tissue replaced by cancer cells after neoadjuvant chemotherapy. However, no viable cancer cells were found and pCR was diagnosed

An 83-year-old Japanese female presented to our hospital 2 weeks after first noticing a lump in the right breast. She had a past medical history of total knee arthroplasty due to osteoarthritis in the left knee, and her level of activities of daily living was poor. Clinical examination revealed a 2 cm, elastic hard lesion with well-defined margins palpable in the lower inner quadrant of the right breast. No lymph nodes were palpable in the right axilla. Mammography revealed a dense mass with partially indistinct margins (Fig. 1a), and ultrasonography showed a well-defined mass with rough borders measuring 21 mm in diameter. Contrast-enhanced magnetic resonance imaging (MRI) showed a highly enhancing lesion on T1-weighted imaging and a high-intensity lesion on T2- and diffusion-weighted imaging (Fig. 1b). The kinetic curve showed a rapid-washout pattern suggestive of malignant disease. Ultrasonography-guided needle biopsy was performed and histopathological examination revealed proliferation of cancer cells with papillary structures, accounting for over 90 % of the whole lesion, supported by an arborizing fibrovascular stroma (Fig. 2a). Invasive papillary carcinoma was diagnosed. Immunohistochemical examination demonstrated Allred scores of 8 for both estrogen receptor (ER) and progesterone receptor (PgR), but a result of 1+ for human epidermal growth factor receptor 2 (HER-2) [2]. Although the breast cancer was considered operable, the patient declined surgery, instead choosing neoadjuvant endocrine therapy with letrozole at 2.5 mg/day. During neoadjuvant endocrine therapy, the tumor gradually shrank despite poor adherence to the regimen due to lack of consciousness of the disease and an incidental complication of liver abscess. She finally consented to breast-conserving surgery after 12 months of letrozole treatment. On preoperative examination, mammography revealed disappearance of the tumor (Fig. 1c). MRI likewise showed no evidence of tumor (Fig. 1d), and ultrasonography showed an area of low echogenicity measuring 7 mm in size, suggesting a treatment scar. Considering her age, breast-conserving surgery was performed without sentinel lymph node biopsy.

Pathological examination showed fibrosis and elastosis, which was thought to represent tissue replaced by cancer cells after neoadjuvant chemotherapy, but there is no viable cancer cells and pCR was diagnosed (Fig. 2b). Considering the age of the patient and her poor level of activities of daily living, adjuvant radiotherapy was not performed, but no recurrence or metastasis had been found as of 13 months postoperatively.

## Discussion

Invasive papillary carcinoma is a rare type of invasive ductal carcinoma, classified as WHO-O code 8503/3 and defined as showing a predominantly papillary morphology (>90 %) in the invasive component. Because of its histological similarity, papillary metastases from other primary sites like lung or ovary carcinoma should be considered in the differential diagnosis [1]. In this case, there was no evidence of metastases from carcinoma at any other primary sites, and primary breast cancer was diagnosed.

In terms of clinical characteristics, Liu et al. [3] reported that invasive papillary carcinoma occurs mostly in the postmenopausal period and is associated with better 5-year overall survival rate and disease-free survival rate than general invasive ductal carcinoma, although the distribution of the four biological subtypes (luminal A, luminal B, HER2, triple-negative) was similar to that of general-type invasive ductal carcinoma.

Generally, when we find papillary lesion in a core needle biopsy specimen, it is sometimes difficult to determine whether the lesion is benign or malignant and to identify the existence of an invasive lesion. Therefore excisional biopsy is recommended for many cases [4]. In the present case, we were able to gather a sufficient volume of specimen by vacuum-assisted large core needle biopsy, making the diagnosis of invasive papillary carcinoma easy. However, we could not reconfirm this diagnosis at surgery, as pCR had been achieved. To the best of our knowledge, this represents the first report of the therapeutic effect of neoadjuvant endocrine therapy for invasive papillary carcinoma.

According to the National Comprehensive Cancer Network (NCCN) guidelines [5], neoadjuvant endocrine therapy for breast cancer may be considered for receptor-positive disease in postmenopausal patients with clinical stage IIA to IIIA, but definitive guidelines are lacking in terms of duration or medication. Clinical practice guidelines published by the Japanese Breast Cancer Society also state that neoadjuvant endocrine therapy may be considered for receptor-positive, HER2-negative disease in postmenopausal patients, but mostly for patients who are unable to undergo or do not consent to surgery [6]. Compared with neoadjuvant chemotherapy, the pCR rate is usually low with neoadjuvant endocrine therapy, but a higher probability of achieving breast-conserving surgery and a lower risk of severe complications may be provided [7]. However, there is no clear relationship between antitumor effects and long-term survival, and further discussion about indications for neoadjuvant endocrine therapy and predictive factors is needed.

The pCR rate is one of the prognostic factors that we can assess outcome in patients undergoing neoadjuvant chemotherapy [8]. However, pCR can be achieved in only a minority of patients with ER-positive disease, irrespective of the chemotherapeutic agents used. Recent consensus papers on neoadjuvant endocrine therapy in breast cancer have indicated that pCR rates ranges from 1  to 8 % in patients with tumors expressing ER [9]. When it comes to neoadjuvant endocrine therapy, the relationship between pCR and overall survival is unclear. Aiming for pCR with neoadjuvant endocrine therapy is thus difficult, and clinicians must try to identify the optimal window for attempting curative surgery while monitoring the therapeutic effect. In terms of the optimal duration for neoadjuvant endocrine therapy, a panel discussion at St. Gallen in 2013 [10] reported that most of the experts believed neoadjuvant endocrine treatment should be continued until maximal response, rather than performing surgery after following a predefined protocol for neoadjuvant chemotherapy. Paepke et al. [11] compared effects between letrozole treatment for 4 and 8 months, finding a significant increase in response rate (57  vs. 90 %, respectively), and noted that longer treatments may allow for an increase in tumor reduction rates. Allevi et al. [12] reported that pCR rate increased up to 17.5 % with 12 months of neoadjuvant letrozole treatment, compared to 5.0 % with 8 months and 2.5 % with 4 months. Prolonged neoadjuvant letrozole treatment is well tolerated with a favorable toxicity profile and results in further tumor volume reduction, and thus may provide incremental benefits to patients for conservative surgery and the induction of a higher rate of pCR. In our case, we considered that pCR was achieved due to the high level of tumor ER expression, and by the long neoadjuvant treatment up to 12 months.

## Conclusion

We report a case of invasive papillary carcinoma in which pCR was achieved after neoadjuvant endocrine treatment. Continued neoadjuvant endocrine therapy with an aromatase inhibitor until the maximal clinical response is obtained may represent the best strategy in neoadjuvant endocrine therapy.

## Abbreviations

pCR: pathological complete response
MRI: magnetic resonance imaging
ER: estrogen receptor
PgR: progesterone receptor
HER2: human epidermal growth factor receptor 2
WHO: World Health Organization
NCCN: National Comprehensive Cancer Network
